# Prevalence of Alexithymia and Associated Factors Among Dental Students in Saudi Arabia: A Cross-Sectional Study

**DOI:** 10.3390/healthcare12212193

**Published:** 2024-11-04

**Authors:** Hebah M. Hamdan, Ghaida Alislimah, Hessa Alshalawi, Khawlah Alharbi, Mohammed I. Alsaif, Ayman M. Sulimany

**Affiliations:** 1 Department of Periodontics and Community Dentistry, College of Dentistry, King Saud University, Riyadh 11545, Saudi Arabia; moalsaif@ksu.edu.sa; 2 Dental Intern, College of Dentistry, King Saud University, Riyadh 11545, Saudi Arabia; ghaida.as999@gmail.com (G.A.); hessa.shalawi@gmail.com (H.A.); 3 General Dentist, Center of Advanced Consultants in Healthcare Education and Training, Riyadh 13216, Saudi Arabia; dr.alharbikhawlaah@gmail.com; 4 Department of Pediatric Dentistry and Orthodontics, College of Dentistry, King Saud University, Riyadh 11545, Saudi Arabia; asulimany@ksu.edu.sa

**Keywords:** alexithymia, depression, dental students, dental education, healthcare education, Saudi Arabia

## Abstract

**Background:** Mental health challenges among university students are pervasive, with alexithymia posing a particularly significant yet understudied challenge. This condition significantly affects an individual’s ability to cope with stress due to difficulties in recognizing, describing, and processing emotions. **Objectives:** This study aims to evaluate alexithymia prevalence and its associated factors among dental undergraduate students and interns enrolled at King Saud University in Riyadh, Saudi Arabia. **Methods:** Data were collected through a self-administered online survey that assessed alexithymia symptoms (using the Toronto Alexithymia Scale [TAS-20]), sociodemographic profiles, lifestyle-related factors, and health-related factors. The associations between participant factors and alexithymia were assessed using chi-square and multiple logistic regression analyses. **Results:** Of the 421 eligible participants, 369 completed the survey (87.6% response rate), revealing a significant prevalence of alexithymia (37.9%). Female gender (AOR = 1.7, *p* = 0.04), depression (AOR = 5.6, *p* < 0.0001), chronic diseases (AOR = 3.5, *p* = 0.003), and childhood abuse (AOR = 2.2, *p* = 0.047) were independent factors significantly associated with alexithymia. **Conclusions:** These findings highlight the pressing need for mental health support within dental education. Early interventions targeting alexithymia could mitigate its adverse consequences, promoting better student well-being and academic success.

## 1. Introduction

Mental health challenges are significant concerns in university education, affecting students across diverse disciplines worldwide. The rigorous academic environment, combined with social and personal stressors, makes university students susceptible to various mental health issues, including depression, stress, anxiety, and sleep disorders [1]. Alexithymia adds to these challenges—a condition that remains relatively understudied yet holds significant implications for an individual’s ability to cope with stress and process emotional experiences [2]. Individuals with alexithymia struggle to identify and express emotions, verbalize feelings, and engage in imaginative processes, leading to difficulties in communication, creativity, and internal emotional exploration [3,4,5,6,7].

The etiology of alexithymia remains incompletely understood, with its development likely influenced by a complex interplay of factors, including early life experiences, sociodemographic characteristics, and health-related conditions [8,9,10,11]. Negative childhood experiences—such as abuse, neglect, or parental separation—are strongly associated with the development of alexithymia [8]. Moreover, research indicates that certain sociodemographic factors, such as lower educational levels, lower income, older age, and single marital status, are associated with alexithymia [9,10,11]. Mental health disorders, including stress [12], depression [9,13,14], anxiety [9,15], eating disorders [16], and substance abuse [17], are also frequently correlated with higher rates of alexithymia. Furthermore, existing evidence suggests that alexithymia is often associated with chronic medical conditions, particularly those involving prolonged pain or stress, such as respiratory conditions [18], digestive diseases [19,20], neuromuscular disorders [21], and diabetes [22].

Alexithymia is estimated to affect approximately 10% of the general population [9,23]. However, research indicates that university students, particularly those in health-related fields, experience significantly higher rates [10,13,24,25]. For example, a study conducted in China found a 37.7% prevalence of alexithymia among medical students [10], while research in Saudi Arabia reported rates as high as 56.5% among medical undergraduates [13,24,25,26]. Although studies focusing specifically on dental students are limited, several factors suggest that this group may be at increased risk. Dental students face substantial stressors, including a demanding academic curriculum, intensive clinical training, and the pressures associated with patient care responsibilities [27]. These challenges may heighten their vulnerability to alexithymia, potentially undermining their emotional competence and impacting their ability to communicate effectively.

Comprehending and communicating emotions proficiently are crucial to psychological well-being and interpersonal connections. In dentistry, emotional awareness is essential for fostering empathy and establishing rapport with patients, which ultimately enhances the quality of care. A lack of emotional awareness and expression may impede these empathetic connections, potentially compromising patient interactions [28]. Studies from various medical fields emphasize the adverse effects of alexithymia on different aspects of individuals’ lives, including interpersonal relationships, mental well-being, and academic performance [29,30]. It has been associated with maladaptive behaviors such as poor self-care, lack of physical activity, smoking, and substance misuse [25,31,32]. These factors can severely impact the overall quality of life, making alexithymia a significant concern for affected individuals [29].

Despite its significance, alexithymia remains relatively understudied among dental students and practitioners. This study aimed to investigate the prevalence of alexithymia and its associated factors among dental undergraduates in Saudi Arabia. This study seeks to foster a deeper understanding of dental students’ well-being and emotional awareness by shedding light on this understudied topic. Ultimately, findings from this study can pave the way for targeted interventions and support mechanisms, alleviating the burden of mental health challenges and enhancing the overall well-being of future dental professionals.

Our research is guided by two specific questions: (1) What is the prevalence of alexithymia among Saudi dental undergraduates? (2) How do various sociodemographic variables (e.g., age, academic level, gender, GPA [Grade Point Average], marital status, parental marital status, type of residence, and household income), lifestyle factors (such as physical activity, smoking, and childhood living arrangements), and health-related factors (including history of psychiatric illness, chronic illness, eating disorders, childhood abuse, and depression) influence the prevalence of alexithymia in this population?

For the first research question, we hypothesize that the prevalence of alexithymia among dental undergraduates will be higher than that of the general population [9,23] and similar to rates reported among students in other healthcare fields [10,13,24,25,26]. For the second question, we anticipate that certain sociodemographic, lifestyle, and health-related factors will be significantly associated with higher levels of alexithymia in the study population.

## 2. Materials and Methods

### 2.1. Study Design and Setting

Using an institution-based, analytical approach, a cross-sectional investigation was carried out involving dental students at King Saud University in Riyadh, Saudi Arabia. Survey data were collected via an online, anonymous questionnaire hosted on SurveyMonkey.com from July to October 2023, with all participants providing electronic informed consent before participation. This research was ethically approved by the King Saud University review board (reference number: KSU-REC E-22-7411). We followed the Strengthening the Reporting of Observational Studies in Epidemiology (STROBE) guidelines to report this study.

### 2.2. Participants, Sample Size, and Sampling

Both male and female dental students from the 1st academic year to intern level were eligible for participation. The necessary sample size was established based on the previous Saudi study which reported a 47% prevalence of alexithymia among medical students [13]. To achieve a 5% margin of error at a 95% confidence level, a minimum of 201 participants were required.

A total population sampling method was employed. The hyperlink to the survey was distributed electronically via email to academic class leaders, who forwarded it to their fellow students through text messaging platforms like WhatsApp (Version 2.23.15.24). Additionally, peer-to-peer communication and word-of-mouth increased the survey’s reach, enhancing the sample’s representativeness.

### 2.3. Study Tool and Measurements

The study questionnaire contained a total of 44 questions under three segments: (i) sociodemographic information, (ii) assessment of alexithymia, and (iii) assessment of depressive symptoms. Approximately 5 min was needed to complete the study questionnaire.

### 2.4. Sociodemographic and Background Information

The questions for obtaining sociodemographic and background information were prepared by the study team based on the literature [10,13,24]. In this section, participants were asked about their age, gender, academic year, grade point average (GPA), marital status, parental marital status, housing, monthly family income, physical activity, and smoking status, and whether they spent their childhood living with their parents.

Additionally, participants were queried about their history of psychiatric illness (any diagnosed mental health conditions), chronic diseases (defined as any long-term medical condition requiring ongoing treatment), eating disorders (conditions characterized by abnormal or disturbed eating habits), and childhood abuse (defined as any form of physical, emotional, or sexual abuse experienced during childhood). These conditions were self-reported, and no diagnostic confirmation was obtained, with each condition recorded as yes or no.

### 2.5. Assessment of Alexithymia Symptoms

Alexithymia was the primary outcome variable of this study, measured using the twenty-item Toronto Alexithymia Scale (TAS-20). The TAS-20 is an extensively utilized and validated tool for assessing alexithymia [33,34]. It includes 20 items divided into three domains: Difficulty Identifying Feelings (7 items), Difficulty Describing Feelings (5 items), and Externally Oriented Thinking (8 items). Responses are scored on a five-point Likert scale, ranging from 1 (strongly disagree) to 5 (strongly agree) [35]. The overall score is calculated by summing the responses to all items, resulting in a possible range of 20 to 100. Participants with scores of 61 or higher were classified as having alexithymia [35,36].

The TAS-20 has been used in multiple studies conducted in Saudi Arabia, where it has shown good internal consistency for the Saudi population [24,37]. In both the original TAS-20 scale [35] and this study, the internal consistency was confirmed with a Cronbach’s alpha of 0.81.

### 2.6. Assessment of Depressive Symptoms

The final segment of the survey evaluated depressive symptoms using the Patient Health Questionnaire-9 (PHQ-9), a validated self-administered tool composed of nine items specifically assessing depression [38]. Items were rated on a scale from zero (“Not at all”) to three (“Nearly every day”). The total score was derived by summing individual scores for items, spanning a range of 0–27 points. A score of 10 or more was considered an indication of depression [39,40]. In the original validation studies, the Cronbach’s alpha for the PHQ-9 ranged from 0.86 to 0.89, indicating good internal consistency [38]. In this study, the Cronbach’s alpha score for the PHQ-9 was 0.86, demonstrating a similar level of reliability.

### 2.7. Statistical Analysis

The analysis of all data was conducted utilizing SAS software, version 9.4. (SAS Institute Inc., Cary, NC, USA). Continuous variables are presented using the mean and standard deviation, while frequencies and percentages are used to report categorical variables. Bivariate analyses, using the chi-square test, were performed to compare participants by alexithymia status and examine related participant factors. Variables that were statistically significant in the bivariate analysis were further analyzed in a multivariate logistic regression model using a stepwise approach. The model’s goodness of fit was evaluated with the Hosmer–Lemeshow test. Statistical significance was set at *p* < 0.05.

## 3. Results

A total of 421 dental students and interns were eligible for this study, with 369 participants completing the survey, yielding a response rate of 87.6%. The participants’ ages ranged from 19 to 25 years, with a mean age of 22.2 ± 1.8 years. Of the respondents, 61.8% were male (*n* = 228) and 38.2% were female (*n* = 141).

Overall, 37.9% of the participants (*n* = 140) were classified as having alexithymia, based on a Toronto Alexithymia Scale-20 (TAS-20) score of ≥61. Additionally, 73.2% (*n* = 270) of participants reported depressive symptoms, with a Patient Health Questionnaire-9 (PHQ-9) score of ≥10. Sociodemographic and background characteristics of this study sample are detailed in Table 1.

In the bivariate analysis, several variables were significantly associated with higher levels of alexithymia. Specifically, significant differences were observed by gender, physical activity, the presence of chronic diseases, childhood abuse, eating disorders, and depression (Table 1). Females demonstrated higher rates of alexithymia compared to males. Moreover, participants who reported lower levels of physical activity; those with chronic diseases, a history of childhood abuse, or eating disorders; and those who experienced depression had higher levels of alexithymia.

To further assess the relationship between these variables and alexithymia, a stepwise logistic regression analysis was performed (Table 2). The goodness of fit of the regression model was confirmed using the Hosmer–Lemeshow test, which indicated a suitable model fit (*p* = 0.5). The results revealed that female participants were 1.7 times more likely to exhibit alexithymia than males (AOR = 1.7 95% CI: 1.03–2.64). Depression was the strongest predictor, with individuals experiencing depression having a 5.6-fold increased likelihood of alexithymia (AOR = 5.6; 95% CI: 2.86–10.89). Furthermore, participants with chronic diseases were 3.5 times more likely to have alexithymia (AOR = 3.5; 95% CI: 1.52–8.04), while those with a history of childhood abuse were over twice as likely to exhibit alexithymia (AOR = 2.2; 95% CI: 1.01–4.60).

## 4. Discussion

Our investigation sought to elucidate the prevalence of alexithymia and its related risk factors among a sample of dental students and interns in Saudi Arabia. The findings reveal a concerning prevalence of emotional difficulties within this cohort, with 37.9% of surveyed dental students exhibiting significant symptoms of alexithymia. This rate is nearly four times higher than the prevalence reported in the general population, which is commonly cited to be around 10% [9,23]. Comparable findings have been documented among healthcare students, both locally and internationally [10,13,24,25,26,41,42]. For instance, Egyptian medical students demonstrated a prevalence of 24.4% [42], while studies among Chinese and Romanian medical students reported prevalences of 37.7% and 36.02%, respectively [10,41]. Within Saudi Arabia, alexithymia rates among medical students have ranged between 26.9% and 56.5%, further underscoring the emotional challenges faced by students in healthcare fields [13,24,25,26].

In addition to alexithymia, our study identified an alarmingly high prevalence of depression, with 78% of participants reporting depressive symptoms. This finding is consistent with previous research on dental and healthcare students. For example, one study reported that 55.9% of Saudi dental students in Riyadh exhibited depressive symptoms [43]. Similarly, another study found that 69.9% of medical and dental students in Saudi Arabia experienced depression [44]. The high rates of alexithymia and depression observed in our study may be attributed to the significant stress associated with the academic journey of dental students. Factors such as high academic demands, fear of failure, time constraints, and social isolation likely contribute to emotional suppression or detachment as coping mechanisms, thereby increasing the risk of both conditions [27,45].

Our analysis identified depression as the most significant risk factor for alexithymia, a finding consistent with earlier research [9,13,14,46]. A prior investigation involving Saudi medical students highlighted a significant association between alexithymia and depression [13]. Furthermore, a meta-analysis reported a moderate correlation between alexithymia scores and the severity of depression, further supporting the observed relationship [14].

The intricate interplay between alexithymia and depression is not yet fully understood; however, theories suggest that a difficulty in recognizing and expressing emotions in individuals with alexithymia may lead to emotional detachment and social isolation, exacerbating depression [14,47,48]. Furthermore, shared neurobiological factors, encompassing altered activity in brain regions responsible for emotional processing, likely contribute to the link between alexithymia and depression. A dysfunction in neural substrates within the prefrontal cortex and amygdala, which are key areas involved in emotional regulation and response, may mediate the association between these conditions [46].

Our study also revealed gender differences, with female students exhibiting a higher likelihood of developing alexithymia compared to their male counterparts. Interestingly, a recent study including 730 high school students in Poland revealed a 31% prevalence rate, with a higher prevalence among female students [49]. While consistent with some previous research [9,26,49], our findings contrast with others [10,25,45], suggesting that the relationship between gender and alexithymia may be influenced by factors such as culture, personality traits, stress, and coping strategies [50]. Further exploration of these underlying mechanisms is warranted to understand this association.

Our findings further demonstrated that participants with a history of childhood abuse faced an elevated risk of alexithymia. This observation aligns with previous studies that highlight the long-term effects of early life trauma on emotional development [8,10,26]. Maltreated children may develop coping mechanisms involving emotional suppression or dissociation, resulting in challenges in identifying and articulating feelings—a hallmark trait of alexithymia [51].

In addition to its connection with mental and psychological issues, alexithymia is also linked to a range of systemic health conditions, such as inflammatory bowel disease [19], neuromuscular disorders [21], coeliac disease [20], and diabetes [22]. Our study further substantiates this correlation, as dental students afflicted with chronic conditions were 3.5 times more susceptible to alexithymia compared to their peers without such conditions. However, it is important to recognize that our assessment of chronic diseases relied on a simple ‘yes/no’ format, which did not distinguish between specific types of chronic illnesses. Plausible mechanisms underlying this association include challenges in emotion regulation that lead to heightened sympathetic arousal and immune impairment [52]. Moreover, alexithymia may promote unhealthy coping mechanisms, such as a sedentary lifestyle, unhealthy dietary habits, and smoking, further exacerbating the risk of chronic conditions [31,53]. While depression, childhood trauma, and chronic conditions emerged as factors associated with alexithymia in our study, it is essential to acknowledge other potential triggers, including anxiety, personality characteristics, attachment style, cultural and societal factors, and genetics, which warrant further investigation [11,54].

This study exhibits some limitations such as the exclusive sample of dental students from one institution, which could affect the generalizability of our findings. Secondly, the cross-sectional design of this study may limit the ability to infer causality. Moreover, this study used self-reported data from participants, which may introduce response bias. Some individuals may over-report or under-report based on social desirability or lack of self-awareness.

Despite these limitations, this study has several potential strengths. Firstly, to the best of our knowledge, this is the first study to assess the prevalence of alexithymia and its risk factors among dental students in Saudi Arabia. Secondly, this study used a validated and reliable index, TAS-20, to assess alexithymia among participants. Additionally, this study explored several sociodemographic and psychological factors associated with alexithymia, contributing to a comprehensive understanding of the emotional well-being of dental students. This could potentially inform targeted interventions and support programs, which could improve the emotional health of dental students in Saudi Arabia.

The high prevalence of alexithymia and depression in our study sample underscores the urgent need for stronger mental health support within dental education. This issue, however, is not exclusive to dental students; it likely extends to other healthcare students, such as those in medical, nursing, and pharmacy programs, who face similar emotional demands and stressors. It is essential for healthcare academic institutions to prioritize mental health programs, counseling services, and workshops aimed at promoting emotional well-being. Special attention should be given to female students and those with chronic illnesses or a history of childhood abuse, as these factors were significantly associated with alexithymia in our study. Creating a supportive learning environment that addresses stressors and fosters resilience is paramount in ensuring the holistic well-being of future healthcare professionals [55].

## 5. Conclusions

Our study highlights a concerning prevalence of alexithymia among dental students and interns, with more than a third of participants experiencing this condition. Factors such as female gender, depression, chronic disease, and childhood trauma were identified as significant correlates of alexithymia. These results emphasize the critical need for improved mental health support in dental education. Efforts to raise awareness about alexithymia and provide targeted interventions can contribute to better overall well-being for dental students and professionals.

## Figures and Tables

**Table 1:** Background characteristics of dental students at King Saud University by alexithymia status (*n* = 369).

Variables	Total Sample	Alexithymia		*p* Value
		No	Yes	
Overall N (%)	369 (100)	229 (62.1)	140 (37.9)	
Age (mean ± SD)	22.2 ± 1.9	22.2 ± 1.9	22.2 ± 1.8	0.90
Gender N (%)				0.0003 *
Male	228 (61.8)	158 (69.3)	70 (30.7)	
Female	141 (38.2)	71 (50.4)	70 (49.6)	
Academic level N (%)				0.95
Pre-clinical	77 (20.9)	47 (61.0)	30 (39.0)	
Clinical	191 (51.8)	120 (62.8)	71 (37.2)	
Intern	101 (27.4)	62 (61.4)	39 (38.6)	
Grade point average N (%) ‡				0.38
4.75–5.00	179 (48.6)	119 (66.5)	60 (33.5)	
4.50–4.75	141 (38.3)	82 (58.2)	59 (41.8)	
4.25–4.50	25 (6.8)	14 (56.0)	11 (44.0)	
<4.25	23 (6.3)	13 (56.5)	10 (43.5)	
Marital status N (%)				1.00
Married	8 (2.2)	5 (62.5)	3 (37.5)	
Not married	361 (97.8)	224 (62.0)	137 (38.0)	
Parental marital status N (%) ‡				0.21
Married	330 (89.7)	208 (63.0)	122 (37.0)	
Divorced/widowed	38 (10.3)	20 (52.6)	18 (47.4)	
Type of Residence N (%)				0.21
Own house	319 (86.4)	202 (63.3)	117 (36.7)	
Rented house/student dormitory	50 (13.6)	27 (54.00)	23 (46.00)	
Family Household income N (%) ‡‡				0.64
<10,000 SR **	41 (11.2)	25 (61.0)	16 (39.0)	
10,000 SR–14,999 SR	48 (13.1)	26 (54.2)	22 (45.8)	
15,000 SR–19,999 SR	61 (16.7)	41 (67.2)	20 (32.8)	
20,000 SR–30,000 SR	75 (20.5)	44 (58.7)	31 (41.3)	
>30,000 SR	141 (38.5)	90 (63.8)	51 (36.2)	
Physical activity N (%)				0.01 *
More than three times a week	82 (22.2)	58 (70.7)	24 (29.3)	
Two to three times a week	81 (22.0)	49 (60.5)	32 (39.5)	
Once a week	72 (19.5)	52 (72.2)	20 (27.8)	
Never	134 (36.3)	70 (52.2)	64 (47.8)	
Smoking N (%)				0.80
Yes	36 (9.8)	23 (63.9)	13 (36.1)	
No	332 (90.2)	205 (61.7)	127 (38.3)	
Living with parents in childhood N (%)				0.79
Yes	351 (95.4)	218 (62.1)	133 (37.9)	
No	17 (4.6)	10 (58.8)	7 (41.2)	
History of psychiatric illness N (%)				0.09
Yes	33 (8.9)	16 (48.5)	17 (51.5)	
No	336 (91.1)	213 (63.4)	123 (36.6)	
History of chronic illness N (%)				0.0002 *
Yes	32 (8.7)	10 (31.3)	22 (68.7)	
No	336 (91.3)	218 (64.9)	118 (35.1)	
History of eating disorder N (%)				0.05 *
Yes	45 (12.2)	22 (48.9)	23 (51.1)	
No	324 (87.8)	207 (63.9)	117 (36.1)	
History of childhood abuse N (%)				0.0007 *
Yes	36 (9.8)	13 (36.1)	23 (63.9)	
No	333 (90.2)	216 (64.9)	117 (35.1)	
Depression N (%)				<0.0001 *
Yes	270 (73.2)	142 (52.6)	128 (47.4)	
No	99 (26.8)	87 (87.9)	12 (12.1)	

* Statistically significant; ** SR = Saudi Riyal; ‡ Missing one participant; ‡‡ Missing three participants.

**Table 2:** Logistic regression analyses of factors associated with alexithymia among dental students at King Saud University (*n* = 369).

Variables	Crude OR (COR)	95% CI	*p* Value	Adjusted OR (AOR)	95% CI	*p* Value
Gender						
Male	Ref	Ref	0.0003	Ref	Ref	0.04
Female	2.2	1.44–3.43		1.7	1.03–2.64	
Depression						
No	Ref	Ref	<0.0001	Ref	Ref	<0.0001
Yes	6.5	3.41–12.51		5.6	2.86–10.89	
History of chronic illness						
No	Ref	Ref	0.0004	Ref	Ref	0.003
Yes	4.1	1.86–8.87		3.5	1.52–8.04	
History of childhood abuse						
No	Ref	Ref	0.001	Ref	Ref	0.047
Yes	3.3	1.60–6.68		2.2	1.01–4.60	

OR: Odds Ratio; 95% CI: 95% Confidence Interval

## Data Availability

Upon request to the corresponding author.
